# *Simplicity DiffExpress*: A Bespoke Cloud-Based Interface for RNA-seq Differential Expression Modeling and Analysis

**DOI:** 10.3389/fgene.2019.00356

**Published:** 2019-05-14

**Authors:** Cintia C. Palu, Marcelo Ribeiro-Alves, Yanxin Wu, Brendan Lawlor, Pavel V. Baranov, Brian Kelly, Paul Walsh

**Affiliations:** ^1^School of Biochemistry and Cell Biology, University College Cork, Cork, Ireland; ^2^NSilico Life Science Ltd., Cork, Ireland; ^3^Laboratory of Clinical Research on STD/AIDS, National Institute of Infectology Evandro Chagas (INI) – Oswaldo Cruz Foundation (FIOCRUZ), Rio de Janeiro, Brazil; ^4^Cork Institute of Technology, Cork, Ireland; ^5^Shemyakin and Ovchinnikov Institute of Bioorganic Chemistry, Moscow, Russia

**Keywords:** differential expression analysis, differential gene expression, statistical modeling, edgeR, transcriptomics, RNA-seq, data-driven

## Abstract

One of the key challenges for transcriptomics-based research is not only the processing of large data but also modeling the complexity of features that are sources of variation across samples, which is required for an accurate statistical analysis. Therefore, our goal is to foster access for wet lab researchers to bioinformatics tools, in order to enhance their ability to explore biological aspects and validate hypotheses with robust analysis. In this context, user-friendly interfaces can enable researchers to apply computational biology methods without requiring bioinformatics expertise. Such bespoke platforms can improve the quality of the findings by allowing the researcher to freely explore the data and test a new hypothesis with independence. *Simplicity DiffExpress* is a data-driven software platform dedicated to enabling non-bioinformaticians to take ownership of the differential expression analysis (DEA) step in a transcriptomics experiment while presenting the results in a comprehensible layout, which supports an efficient results exploration, information storage, and reproducibility. *Simplicity DiffExpress’* key component is the bespoke statistical model validation that guides the user through any necessary alteration in the dataset or model, tackling the challenges behind complex data analysis. The software utilizes *edgeR*, and it is implemented as part of the Simplicity^TM^ platform, providing a dynamic interface, with well-organized results that are easy to navigate and are shareable. Computational biologists and bioinformaticians can also benefit from its use since the data validation is more informative than the usual DEA resources. Wet-lab collaborators can benefit from receiving their results in an organized interface. *Simplicity DiffExpress* is freely available for academic use, and it is cloud-based (https://simplicity.nsilico.com/dea).

## Introduction

OMICs techniques open the doors to researching organisms from a comprehensive perspective, enabling the exploration of the intricate network of relationships, as opposed to analyzing point biological variations. The scaling up of the analysis enabled the understanding of many of the molecular aspects behind an organism’s features, while at the same time revealed that the mechanisms involved in the control of transcription, translation and the organism physiology, in general, are more complex than once thought. Therefore, it is no surprise that OMICs research requires the effort of multi-disciplinary teams and it is quite common to see a publication with more than ten co-authors. From this new perspective, many challenges arise, one being the knowledge transfer and communication between professionals with different backgrounds. Other challenges are well explored, such as analysis and storage of complex data, with every new technique for high-throughput molecular biology research requiring new methods for data analysis and interpretation (Finotello and Di Camillo, 2015; Han et al., 2015; Yuryev, 2015; Byron et al., 2016; Conesa et al., 2016).

Among the OMICs techniques, transcriptomics rapidly became a popular methodology for profiling gene expression through RNA-seq (Nagalakshmi et al., 2008). Transcriptomics can be applied to the analysis of messenger RNAs, non-coding RNAs (such as long non-coding RNAs, microRNAs, and transfer RNAs), the investigation of mRNA isoforms and can be combined with other methods to enhance analysis (Byron et al., 2016; Conesa et al., 2016). Originally, RNA-seq techniques were developed for sequences from pooled cells, which is known as “bulk RNA-seq.” Later on, single-cell RNA-seq methods were developed, requiring not only new laboratory procedures but also the development of novel approaches to process and analyze the data (Tang et al., 2009).

The relative abundance of the set of RNAs found in a sample reflects the level of expression of the corresponding genes, indicating the cells’ state and the aspects involved in the determination of a certain condition (Finotello and Di Camillo, 2015). The objective of DEA is to identify the mRNAs (or other transcribed sequences) that have changed significantly in abundance across treatment groups in an experiment. A typical DEA based workflow firstly requires the mapping of the sequenced reads of each sample to a reference genome or a transcriptome (when available). The following step is the estimation of how many reads matched to different loci or transcripts, the organization of the retrieved information in the “read-count table,” and finally the completion of the necessary corrections such as distribution and coverage normalization. All of the above steps must be done using quality checkpoints, and the analysis strategies may vary according to the organism being studied and the research objective (Oshlack et al., 2010; Conesa et al., 2016).

Summarizing the sequenced data into a read-count table presents important challenges and, on top of that, only precise and powerful tests can efficiently detect the differential expression (Oshlack et al., 2010; Finotello and Di Camillo, 2015; Han et al., 2015). Regardless of the challenges involved in the read-mapping step to generate the read-count table, it was shown that most tools that run this step perform equally (Costa-Silva et al., 2017). On the other hand, the methods applied to DEA have the greatest influence on the final results, and no current strategy offers optimum results (Costa-Silva et al., 2017). Therefore, the real challenge is to identify which transcripts are affected by the phenomena targeted by the research (treatment, cell types, etc.), among all the observed expression changes. Moreover, this is highly dependent on the accurate modeling of technical and biological variability (Finotello and Di Camillo, 2015).

It is undeniable that there is a heavy demand on the bioinformatics skills needed to process the high-throughput sequencing raw data files and the subsequent statistical skills to apply the methods that can uncover the relevant features in the data. A common mistake is to assume that the transcriptomics analysis ends with the list of genes differentially expressed, whereas it is more likely to lead to the next stage of the research. The research team still needs to explore the biological meaning behind the data analysis results, carry out gene set enrichment analysis or similar strategies, retrieve literature to support understanding the biological context and, ideally, test hypothesis by carrying out new wet-lab experiments (Han et al., 2015).

*Simplicity DiffExpress* tackles the statistical analysis steps that are required after the raw RNA-seq data is summarized in read-count tables. The main objective is to improve discovery by facilitating the statistical modeling of the DEA, with no programming skills required. It also offers an interactive interface with guided steps and the presentation of the results in a practical and shareable interface. These features are critical in the study of complex biological questions where multiple factors define the observed phenotype. *Simplicity DiffExpress* opens the doors to non-bioinformatician researchers to explore the data, and we believe it improves the discovery process by enabling the person who knows best about the biological aspects to be hands-on with the statistical analysis without an intermediary bioinformatician. Nonetheless, bioinformaticians benefit from the validation feedback and the practicality of results reproducibility that can be re-visited in any time-point and shared.

## Methods

### Interface Implementation

**FIGURE 1:**
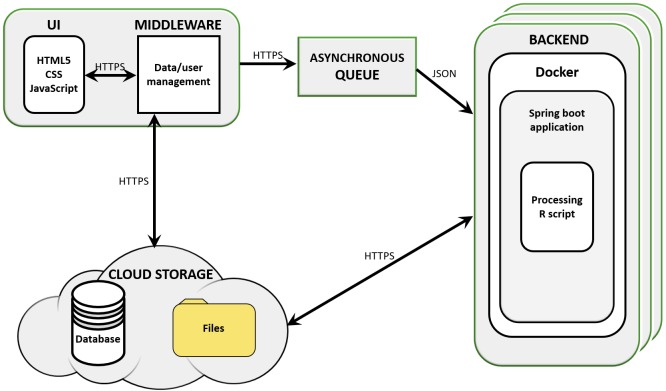
*Simplicity*’s cloud-based structure. *Simplicity* is available on-line and presents a user interface (UI) that carries out real-time processing through its middleware storage and scripts. User requests are managed by an asynchronous queue and data is passed to cloud storage. Once a processing node is available, the backend retrieves the analysis parameters and data from the cloud storage and analyses it, delivering results to the user and compiling reports and interactive visualizations in the UI.

The workflow was implemented as part of *Simplicity*^TM^, a cloud-based software designed for supporting bioinformatics services to non-bioinformaticians (Walsh et al., 2013). Simplicity workflows’ architecture is built using a combination of JavaScript, .NET, Java, and Python based components, which implement the UI, middleware, message queue, and storage (Azure Blob, Figure 1). *Simplicity’s* UI is implemented in HTML5, JavaScript, and CSS. In the case of *DiffExpress* input, .NET also submits the data to R scripts in the middleware to run on-the-fly validations. The communication interface is made up of the queue system and storage elements. The queue system was developed in Java and Spring Boot and runs in a Docker container, controlling which jobs and data are sent to the backend to be processed. In the backend, an agent written in Java interacts with the queue, the storage and the service, which runs in a Docker container on a Linux server host. The resulting output files, when complete, are uploaded back to storage. During the processing, a JSON file containing information on the pipeline progress is constantly updated into the storage. Once this JSON file signals that the job was completed, an email is sent to the user. After he/she securely authenticates his/her login credentials in *Simplicity*, the user is granted access to the results interface, implemented using the same strategy as the input interface. The communication interface pulls the output results from the storage and presents them.

#### Data Validation

The first validation step is to check if the sample names on both read-count and metadata table match and to remove unwanted characters from the labels (implemented on .NET). Missing data (“NA”) is retrieved using R scripts and is with dealt by removing samples or transcripts. The model fitness test is written in R and first evaluates if there are enough degrees of freedom, then, it applies QR decomposition to the statistical model design matrix to verify if it is full rank (all rows and columns are linearly independent) and, finally, checks if there are at least two samples for all the factor combinations generated by an interaction. The user is always informed of any detected issue and, when possible, offered an option on how to deal with it.

### Differential Expression Analysis Implementation

The DEA of *DiffExpress* is fully implemented on Ubuntu 16.04.4 LTS, R version 3.5.2 (R Core Team, 2017), and based on *edgeR* version 3.22.5 (Robinson et al., 2010; McCarthy et al., 2012). *EdgeR* and *DESeq2* (Love et al., 2014) are among the best DEA performers (Finotello and Di Camillo, 2015), enabling multi-group comparisons (Oh et al., 2014), and, in our experience, *edgeR* offers the best approach to model complex data, therefore it was chosen to be the basis of our workflow. We use the library *jsonlite* version 1.6 (Ooms, 2014) to recover the analysis parameters passed as JSON files and *pheatmap* version 1.0.12 (Kolde, 2012) to generate heatmaps. The DEA scripts were initially tested on a dataset investigating changes in the modulation of rat small non-coding RNA due to exercise intensity, which required the modeling of a continuous variable (Oliveira et al., 2018). The environment information with the updated version of the libraries and programs used are presented on the results report, allowing the user keep track of upgrades done in the future.

#### Input Files

*Simplicity DiffExpress* requires two tables as input, which can be a CSV or TXT file. The interface provides options to set the parameters to read the files and on-the-fly visualization of how the data is being processed, enabling flexible input format. The current files’ size is unlimited.

The first table to be uploaded is the read-count table which presents the raw read counts mapped to each genomic tag (genes). There are no requirements regarding transcript IDs formats, although they must be presented in the first column of the file. The remaining columns should be numeric (with the sample name as heading). It is a requirement that the data is not transformed because *edgeR* automatically takes into account the total size (total read number) of each sample/library in all calculations of fold-changes, concentration, and statistical significance. In other words, RPKM, FPKM, and TPM -transformed data are not compatible (Robinson and Smyth, 2008).

The second table contains the metadata and must have (1) a row for each sample/library in the count table; (2) a column for each variable(s) of interest. *Simplicity DiffExpress* automatically removes any sample that is not present in both tables (the user receives a warning). The metadata table may contain any relevant information to understand the data, such as phenotypic features, clinical outcomes or experimental information (such as collection day, batch, institution). Later, the user will inform which of the information will be used in the statistical design, therefore there is no issue if the table contains variables beyond the ones that are intended to be used in the analysis.

#### Low-Count Filtering

A dataset usually has thousands of genomic features, and not all of them have enough reads to contribute to the DEA. In addition, these low counts may interfere with some of the statistical methods used in the pipeline. Therefore, it is strongly recommended to filter them out prior to further analysis. Nonetheless, the user can either opt to not filter out low counts or to decide what is the minimum CPM that a genomic feature must have in order to be kept in the analysis.

#### Normalization

In *Simplicity DiffExpress*, normalization is a mandatory step. The dataset is normalized for RNA composition by trimmed means of *M*-values (Robinson and Oshlack, 2010), which is the default methodology implemented on *edgeR*. The normalization step adjusts the RNA composition effect, avoiding the issue that the remaining genes falsely appear to be down-regulated in that sample/library.

#### Dispersion

The genomic features dispersion estimation is necessary so that it is consistent across replicates and in *Simplicity DiffExpress* it is based on the weighted likelihood empirical Bayes method (Robinson and Smyth, 2007). *Simplicity DiffExpress* uses *edgeR*’s Cox-Reid profile-adjusted likelihood method for all genomic features. It fits a GLM from an informed design matrix, allowing for all systematic sources of variation to be accounted for in the estimations (McCarthy et al., 2012; Chen et al., 2014; Oh et al., 2014). In addition, the user may decide whether the analysis should be robustified against potential outliers.

#### GLM Fitting

Once the above steps are completed, a Negative Binomial GLM is ready to be fitted to the dataset, as described by McCarthy et al. (2012). It conducts a gene-wise statistical test for a given coefficient or coefficient contrast of the variable(s) of interest.

#### Likelihood Ratio Test for the Selected Variables

This method is applied to test the ratio of deviances between nested models with and without the estimation of coefficients or coefficient contrast of the variable(s) of interest in the Negative Binomial-GLM model, respectively. It is at this s*tag*e of the analysis that genes differentially expressed between groups/conditions are actually identified and the gene-wise *p*-values are corrected for multiple comparisons using the Benjamini-Hochberg false discovery rate method (Hochberg and Benjamini, 1990).

### Case-Study

The public dataset GSE68086^1^ for the use case, was originally published by Best et al. (2015). This dataset consists of RNA-seq data of 283 blood platelet samples obtained from 228 patients with six types of malignant tumor and 55 healthy donors. The large number of samples and the availability of metadata allows for a great data modeling opportunity. The statistical model used was *“∼ cancer + Metastasis + batch + Gender + Age”* for estimation of cancer and Metastasis effect, respectively. In both models, *batch*, *Gender*, and *Age* were included as confounders to minimize sample bias in the estimations of interest associated with variables *cancer* and *Metastasis* (Supplementary Figure S1).

Not all series on GEO are suitable for DiffExpress since there are assorted types of data that can be available. For GSE68086, the read-count table was available as Supplementary File and the metadata was obtained from the “Series Matrix” file. It was necessary to explore the “Series Matrix” file, select relevant information, such as the sample IDs that matched the read-count table, batch dates, cancer type, age, and gender. Some further formatting was done to remove the field names from the table cells (e.g., “*cancer type: BrCa*” became “*BrCa*”). The current series publicly available on GEO no longer provides information on *Age*, *Gender*, and *Metastasis*.

## Results

In this section, we do an overview of the features provided by the interface and present a case study using the public available dataset retrieved from GEO under the series identification GSE68086 (see text footnote 2), containing the RNA-seq data of 283 blood platelet samples obtained from 228 patients with six types of malignant tumor and 55 healthy donors (Best et al., 2015). The dataset size and metadata availability offered a great opportunity to test different statistical models. More information on the implementation and how to use *Simplicity DiffExpress* are provided in the documentation^2^, tutorial page^3^, and video^4^.

### Input Interface

#### Format-Flexible Data Input

**FIGURE 2:**
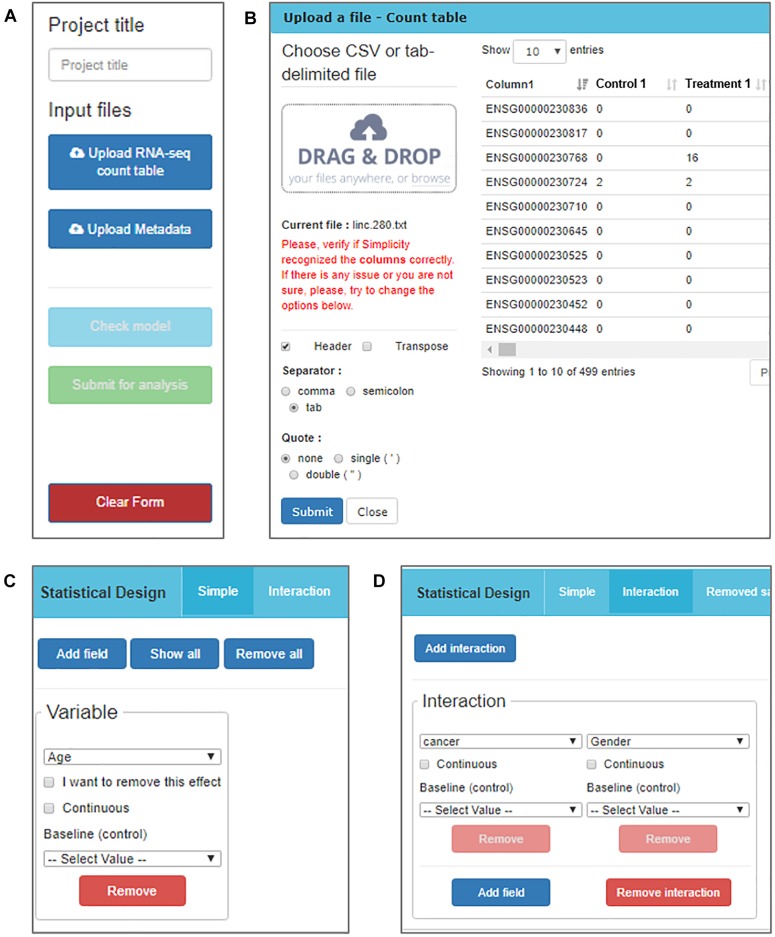
*DiffExpress* input options. **(A)** The user is initially required to input a project title and upload two text files containing the read counts and metadata. **(B)** The upload window displays the table being uploaded in real-time allowing for verification if the interface is reading it correctly. The options on the left side can be altered to adjust the file-reading. **(C)** Once the tables are uploaded, a menu to include variables in the statistical model is enabled. The user should inform what is the baseline between the categories of a factorial variable or mark it as continuous. **(D)** It is also possible to study the interaction between two or more variables.

The RNA-seq data must be processed and organized in a read-count table in order to be analyzed in *Simplicity DiffExpress*. The originally observed expression counts are required, so no data transformation is necessary prior to analysis, once the workflow will handle it subsequently (Robinson et al., 2010). A second table, called “metadata table,” containing the features that characterize the samples must also be provided since it will be used to define the samples groups. The file upload buttons are the first features made available to the user once they access the platform (Figure 2A).

*Simplicity DiffExpress* was designed to deal with variable table formats, and the user can inspect how the software interprets their files on the go and can change the settings as required (Figure 2B). One of the key features of this platform is to allow the user to define the statistical design that best represents the experiment. *Simplicity DiffExpress* offers a form where the user should pick at least one of the features in the metadata table and fit a statistical model (Figure 2C,D). It is also recommended that the sources of bias are informed when defining the statistical model in the input interface. By including all known sources of undesired bias (e.g., batch effects) in the statistical model, the data analysis will take into consideration all those factors which will provide more precise estimations. Supplementary Figure S1 exemplifies how the statistical model used in this work was set-up.

#### On-the-Fly Validation

In order to make the DEA on *Simplicity DiffExpress* more accessible, a graphical UI was implemented to provide clear and immediate feedback for the user. Therefore, the validation steps are key features of the software, to ensure that the data are adequate before submitting for the full analysis. The software informs the user if there are any missing value (represented by “NA”), any mismatch between samples IDs, and checks if it is possible to fit the model (described in “Methods”). This is performed by calling specialized validation R routines (R Core Team, 2017) in the *Simplicity DiffExpress* software validation module, implemented in the middleware. If any issue is identified, the interface presents possible solutions to the user (Supplementary Figure S2A) and specifies the variables that are causing it (Supplementary Figure S2B). In other words, the validation module secures the chances of a successful run of the DEA prior to submitting data to the server.

#### Other Features

All features in the input interface have a short user guide displayed on the bottom of the page, known as the “*Step Wizard*” (not shown). This area is designated to provide a brief overview on which options are available and which actions the user is expected to take. Moreover, some parameters can be customized, and they are made available at the “*Statistics*” tab (Supplementary Figure S3A).

Furthermore, when the validation procedure detects modeling issues, *Simplicity DiffExpress* will offer the option to resolve them by either removing a variable from the model or by eliminating some samples. It is important to highlight that the interface always restores the samples when changes in the statistical model resolve the issue. To allow the user to keep track of all the tested models and adjustments made, the interface provides the “*Removed samples*” tab (Supplementary Figure S3B), which is dedicated to specific details of samples that were eliminated from the current model and a “*History*” tab (Supplementary Figure S3C) that enlists all models tested.

Once all information is provided, and the statistical model has passed the fitness test (*“Check model”* button on Figure 2A), the analysis can be submitted. The user may finish the session at this point or choose to keep using the uploaded data, an option offered to facilitate the creation of new statistical models for the same dataset. Meanwhile, once the workflow management receives the job request, the analysis can take from a couple of minutes to a few hours, depending on the complexity of the statistical models and the dataset size. Once it is finished, the user will receive an email informing that the results are ready to be accessed.

### Results Interface

The *Simplicity* system presents a list of all pipelines run by the user, highlighting the completion status and submission date (Supplementary Figure S4). The users may grant access to a pipeline to specific researchers of their choice, and this access can be revoked at any time by the user. Although the results are sharable, the invitees do not have access to the original input files. This feature favors collaborative work by enabling the whole team to explore the results in an organized presentation. Additionally, it supports reproducibility since all information regarding the analysis parameters is documented and stored at *Simplicity* and permanently linked to the pipeline.

**FIGURE 3:**
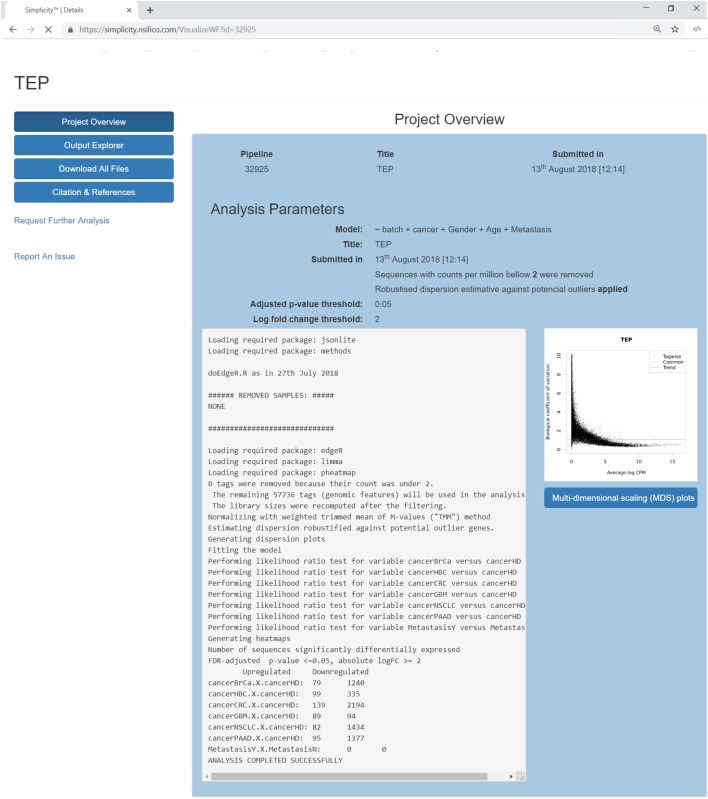
Project’s results overview interface. The blue buttons on the top left allow the user to navigate through the results features, it is also possible to report issues or request further analysis. The main panel reviews the analysis settings and its log, which lists all the steps done during the pipeline run and generates a summary of the genes found up and down-regulated. On the right, a dispersion plot showing the data distribution based on the biological coefficient of variation log CPM average. The biological coefficient of variation represents the coefficient of variation that would remain between biological replicates if sequencing depth could be increased indefinitely. Finally, the user can explore the generated multidimensional scaling plot clicking on the button on the right.

**FIGURE 4:**
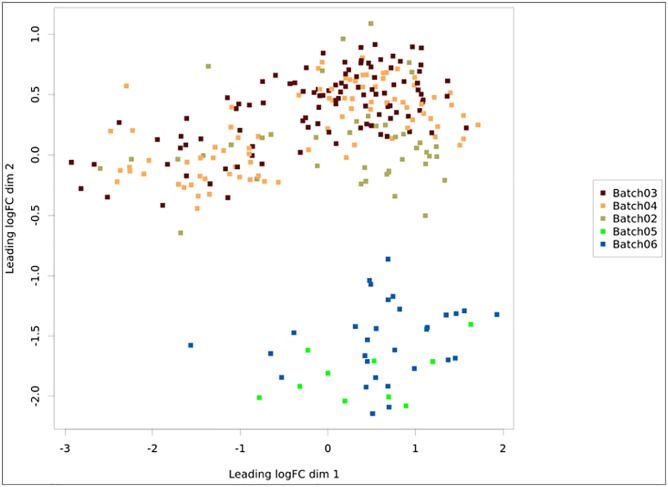
Multidimensional scaling plot with the samples colored according to batch. The *x*- and *y*-axes represent the leading log-fold-changes between each pair of RNA samples, which is given by the root-mean-square of the largest absolute log-fold-changes between each pair of samples. In this example, there is a batch bias, whereas samples from batches 2, 3, and 4 are separated from 5 and 6.

Once a pipeline result is chosen, the user is brought to a new page enlisting all information regarding the DEA (Figure 3), including the chosen setting and analysis log. The log describes all the steps carried on the analysis and summarizes how many genes were found differentially expressed. It also provides access to the biological coefficient of variation plot and multidimensional scaling plots. The later plot presents the leading log-fold-change between each pair of samples and supports identifying structure and heterogeneity in the relative expression data (Figure 4). In the example presented here, it is possible to notice that there is a data structure due to batch. The buttons on the left (Figure 3) offer further functionalities, such as exploratory analysis of the results (“*Output Explorer*”), download all results, information on how to cite the *Simplicity DiffExpress* methods and the possibility to contact support.

**FIGURE 5:**
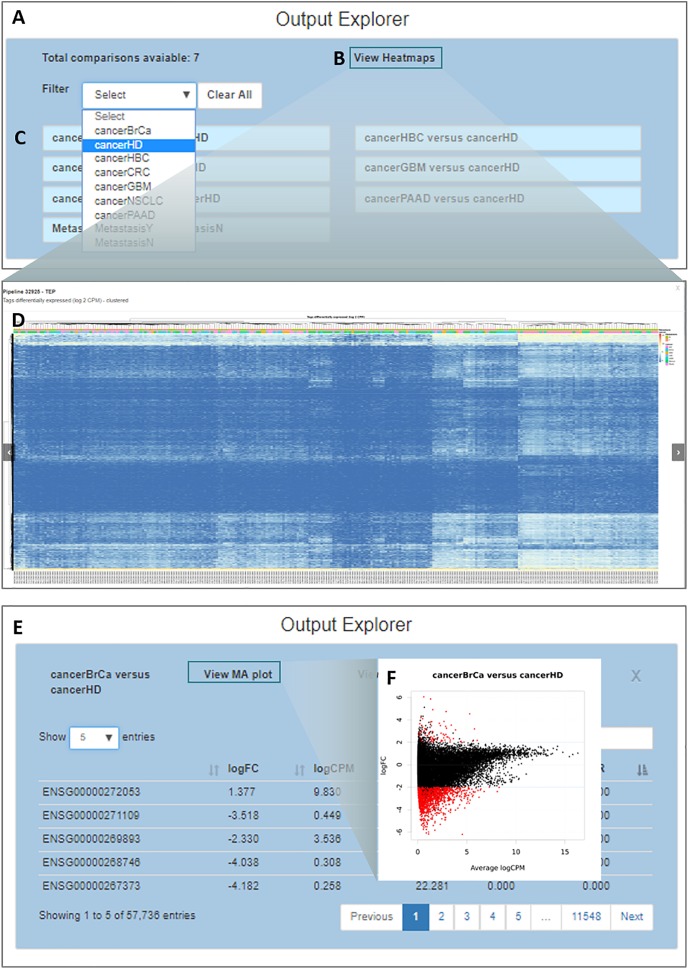
Output explorer options. **(A)** The initial window where **(B,D)** heatmaps can be accessed and **(C)** listing all comparisons across variables. It is possible to filter the comparison list based on the variable category. **(E)** Results of a selected comparison (in this case BrCa vs. HD – breast cancer versus healthy donor). **(F)** An MA plot displaying the log (base 2) fold-change observed for the average log CPM of each group of interest (e.g., BrCa and HD), with genes differentially expressed highlighted in red.

By clicking on the “*Output Explorer*” button, the users have access to a window (Figure 5A) containing heatmaps and providing an overview of the data (Figure 5B,D) and a list with all comparisons between variables done in the DEA (Figure 5C). Once a comparison is chosen for further exploration, they are taken into a page where the results table is displayed (Figure 5E). In the case where more than two transcripts are differentially expressed, a specific MA plot (Figure 5F) and heatmaps are made available to enable an exploratory analysis of the results.

*Simplicity DiffExpress* will generate multiple tables with the DEA results. The number of tables depends on (1) the variables included in the statistical design; (2) the number of levels which each of the categorical/nominal variables has (e.g., in our case-study, variable *cancer* has seven levels: healthy donor and six cancer types); and, (3) if the user sets the program to carry out DEA between every level of the categorical/nominal variables or only contrasts the levels against the baseline. The researcher should interpret the differential expression significance based on the chosen false discovery rate; by default, it is set as 0.05.

Furthermore, it is recommended that the user follow the citation guidelines to ensure all credit is correctly presented; all information is available at the “*Citation and References*” button. Finally, results are restricted to the user and available upon login, and all images and tables can be saved locally through the button “*Download All Files*” (Figure 3).

## Discussion

**FIGURE 6:**
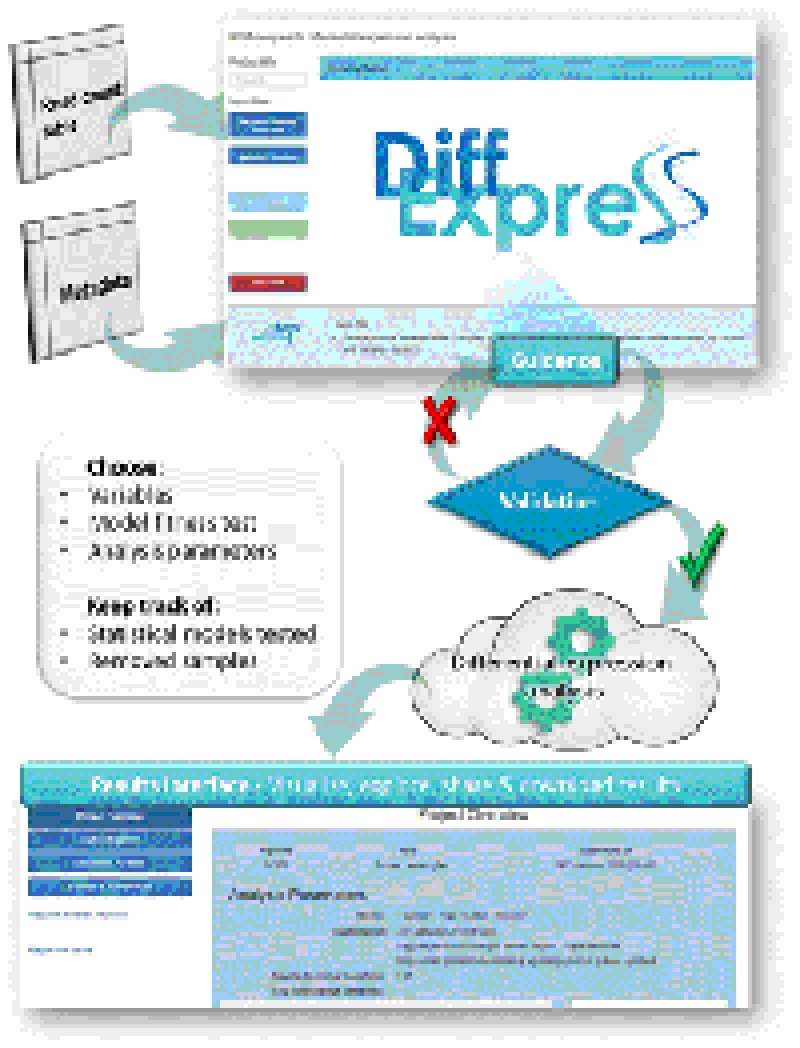
Overview of the *DiffExpress* workflow. The web-interface requires a read-count table and a metadata as input. The users will set-up their analysis which will undergo a real-time validation prior to submission for analysis. If any issue is encountered, the interface will guide the users through the possible actions. Once the statistical model is successfully fitted, the pipeline can be submitted. The data will be sent to the cloud and once the analysis is finished, the users can retrieve and explore the results in a secondary web interface, linked to their accounts.

The primary objective of *Simplicity DiffExpress* is to allow researchers with or without prior bioinformatics knowledge to create DEA models in order to study quantitative changes in gene expression levels between experimental groups. *Simplicity DiffExpress* achieves this through a user-friendly, intuitive, flexible and interactive cloud-based platform (Figure 6). The platform also provides clarity, real-time answers, and data validation. *Simplicity DiffExpress* is available at https://simplicity.nsilico.com/DEA, and it is free for academic use.

In the context of RNA-seq DEA, there are two major types of experimental designs: (1) pairwise group comparisons, where the samples were collected in a single time point and targets differences across two or more biological groups; and, (2) progression experiments, aiming to characterize the dynamics of a biological phenomenon (Oh et al., 2014). Time-series are the most common examples of the progression experiments, where the samples are collected in different points over a time window, but they can also relate to analyses of samples submitted to different intensities of interventions, such as drug dosages. Ideally, the experimental design should account for other sources of nuisances, such as different batches, age, sex, and replicates (Oh et al., 2014; Han et al., 2015). Controlling the sources of variation when designing and modeling correctly all these factors reflects directly in the capability of successfully identifying differentially expressed sequences. Therefore, it is critical to understand those variables, correctly identifying if they are continuous or categorical and how they relate to each other (Is there an interaction effect? Are they independent?). *Simplicity DiffExpress* tackles this challenge providing support to modeling both continuous and categorical variables, regardless of how many levels a category can have, enabling interaction analysis while providing feedback on issues preventing the statistical model fitting.

For example, in the work by Oliveira et al. (2018) rats were submitted to low, moderate and high-intensity treadmill protocols to investigate the impact of exercise on serum extracellular vesicles and their small RNAs. If the exercise intensity was modeled as a factor of four levels (“no exercise,” “low,” “moderate,” and “high” intensities), the experimental design would be misrepresented because the intensity levels would be interpreted as unrelated treatments. What should be done instead, is to include the average treadmill speed applied to each group and modeled it as a continuous variable, enabling to capture potential gradual expression changes in relation to the speed. Going back to the analysis of the blood platelet samples, in Figure 3 we can observe that no transcripts related to *Metastasis* were found differentially expressed. This is likely because the metastasis features and onset changes depend on the cancer type, therefore a better model would include an interaction between the variables *Cancer* and *Metastasis*.

*Simplicity DiffExpress* core analysis is based on the well-known and broadly used resources offered by the R (R Core Team, 2017) package *edgeR* (Robinson et al., 2010; McCarthy et al., 2012). *Simplicity DiffExpress* makes the valuable *edgeR* features available to a non-bioinformatician public and augments the use of *edgeR* with key validation support, used to identify issues in the dataset and statistical model prior to running the analysis. This is a crucial feature since, when running a script for an *edgeR*-based analysis, many errors are only identified after some time is elapsed, therefore a strong validation is a valuable contribution toward the analysis process. Moreover, the technical aspects of the analysis (input format, validation issues, statistical parameters) are presented in clear language in order to make it accessible to non-specialists. All these features are combined with detailed documentation, which includes insights into the statistical aspects of the analysis and a step-by-step tutorial.

In comparison to other web applications that provide DEA for the user without programming experience, like DEApp (Li and Andrade, 2017) and DEBrowser (Kucukural et al., 2019), the key advantages of *Simplicity DiffExpress* are related to input files and complex data modeling. *Simplicity DiffExpress* has no limit for file size and offers clearer feedback regarding issues when reading the files and incompatibilities between count-table and metadata. To our knowledge, *Simplicity DiffExpress* is the only platform of this type that allows the analysis of variables as continuous, which is very important as explained above, and it offers more flexible options to define interactions for multi-factorial analysis because the interactions are not mandatory and can be done with specific features. Moreover, both DEApp and DEBrowser require the user to inform manually each paired comparison to be studied, which can be not practical when dealing with many variables or categorical variables with many levels. In summary, *Simplicity DiffExpress* structure is more robust to deal with datasets with complex metadata, besides the fact it is able to store and share the results.

*Simplicity DiffExpress* can be used on a broad range of data sources, as long as the RNA-seq data is summarized in the read-count table, without any transformation. The investigation of complex biological outcomes will greatly benefit from *Simplicity DiffExpress* features. For example, RNA-based measurements can be applied across diverse areas of human health, including disease diagnosis, prognosis, and therapeutic decisions. At the moment, it supports clinical practice for infectious diseases, cancer, transplant medicine, and fetal monitoring (Byron et al., 2016). *Simplicity DiffExpress* features offer useful assistance for health-care because it provides functionality for guiding users on modeling multi-factorial and temporal designs. When dealing with cohort studies there are many bias sources beyond the obvious genetic variability across individuals. By enabling investigators with clinical knowledge to run their own DEA, our software increases the possibilities of discovery because users can combine variables, correct for sources of bias and test hypotheses themselves and at their convenience since they no longer depend on an intermediary researcher between them and the analysis. It can also be used as a means to support the communication between bioinformatician and wet-lab researchers because it presents the data in a user-friendly set-up.

Differential expression analysis can generate a high number of outputs depending on the experimental design. *Simplicity DiffExpress* also addresses file management issues by saving the analysis parameters and organizing the output files systematically. This feature supports research reproducibility and reporting. Moreover, the *sharing* feature facilitates the exchange between collaborators, avoids e-mail clutter and promotes transparency.

## Concluding Remarks

*Simplicity DiffExpress* aims to support the research of differentially expressed sequences by providing an intuitive interface with guidance through the steps and, on overcoming data modeling issues. Another critical advance provided by *Simplicity DiffExpress* is the data validation: besides checking the correspondence between samples IDs in the input files, it tests the statistical model fitness prior to the DEA enabling the immediate identification of any issues in the design and indicating solutions for it. This feature advances the functionalities provided by the R library *edgeR* (Robinson et al., 2010; McCarthy et al., 2012). Moreover, the results interface was designed to present the outputs of the DEA in an organized and easy to navigate format, addressing an issue regarding files management that can be critical since, depending on the experimental design, the output results can be extensive.

## Author Contributions

CP conceptualized the study, performed statistical analysis, validated the software, and wrote the original draft. MR-A conceptualized the study, performed statistical analysis, wrote, reviewed and edited the manuscript. YW performed software validation. BL performed software validation, and reviewed the manuscript. PB conceptualized the study, supervised, reviewed, and edited the manuscript. BK conceptualized and supervised the study, and reviewed the manuscript. PW conceptualized the study, performed software validation, supervised the study and reviewed the manuscript.

## Conflict of Interest Statement

CP, YW, and BLwork in collaboration with the company NSilico Life Science Ltd. BK is the CEO and PW is the CTO of the company NSilico Life Science Ltd. The remaining authors declare that the research was conducted in the absence of any commercial or financial relationships that could be construed as a potential conflict of interest.

## Footnotes

1 https://www.ncbi.nlm.nih.gov/geo/query/acc.cgi?acc=GSE68086

2 https://simplicity.nsilico.com/Home/Document

3 https://simplicity.nsilico.com/Home/Support

4 https://www.youtube.com/watch?v=QKZu46c4HfU

## Abbreviations

CPM: counts per million
DEA: differential expression analysis
GLM: generalized linear model
UI: user interface
