# Supplementary material for: Simplicity DiffExpress: A Bespoke Cloud-Based Interface for RNA-seq Differential Expression Modeling and Analysis
Source: Front Genet. 2019 May 14;10:356. doi: 10.3389/fgene.2019.00356 (PMC6527599; doi:10.3389/fgene.2019.00356)
Supplement: Supplementary file 1 [file Data_Sheet_1.pdf]

Supplementary Material

DiffExpress: a bespoke cloud-based interface for RNA-seq differential expression modeling and analysis

Cintia C. Palu*, Marcelo Ribeiro-Alves, Yanxin Wu, Brendan Lawlor, Pavel V. Baranov, Brian Kelly, Paul Walsh*

*** Correspondence:**
Cintia Palu
cintia.palu@nsilico.com
Paul Walsh
paul.walsh@nsilico.com

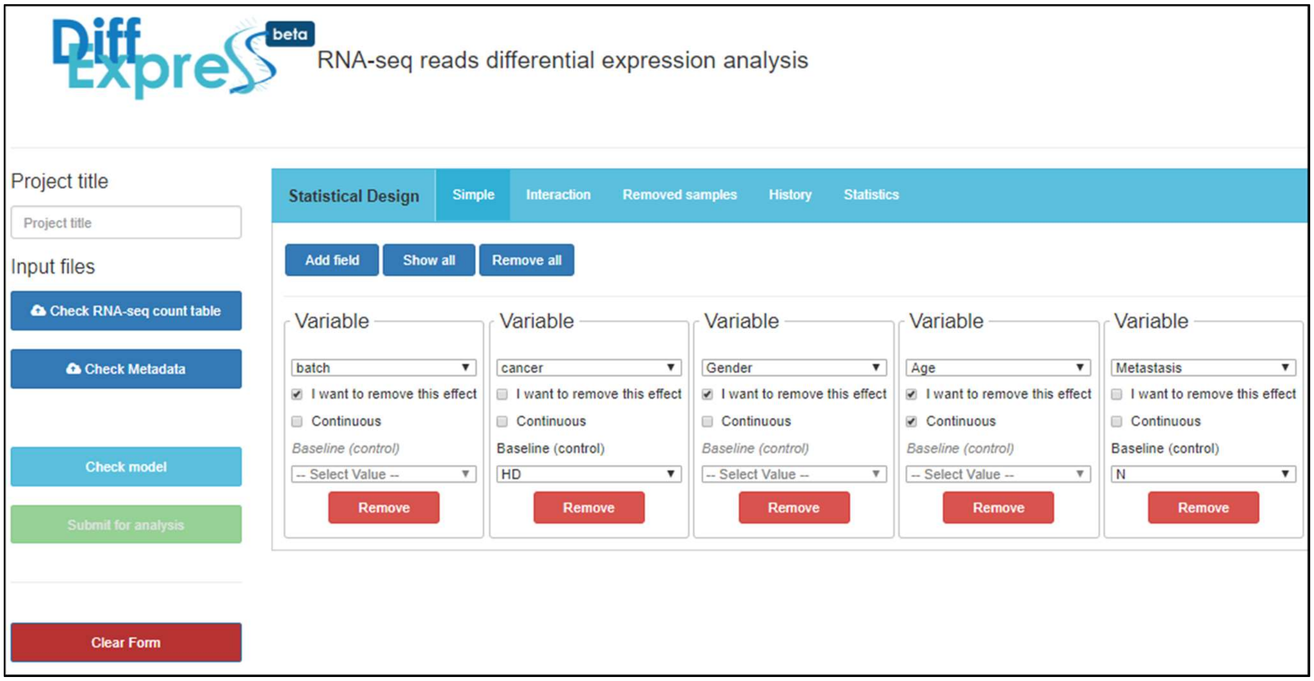

Supplementary Figure 1. Statistical design modeling. It shows the set-up used for the analysis presented as an example in the paper (model '~ *batch* + *cancer* + *Gender* + *Age* + *Metastasis*'). In this example, the focus is to look for genes differentially expressed in the samples from six cancer types and look for genes that could explain the metastasis occurrence. *Batch*, *Gender* and *Age* are considered bias sources and should be in the statistical model, but there is no need to test if there are differentially expressed genes due to those variables. Therefore the option 'I want to remove this effect' is selected for the variables. It is also important to inform which variables are continuous like was done *Age.* In the case of categorical variables that are not a source of bias, it is necessary to inform which level is the baseline. In the case of *cancer*, the aim is to identify changes in transcripts in relation to healthy donors, identified as '*HD*' in this dataset. For *Metastasis* the baseline is '*N*' because it indicates that no metastasis was observed in the patient. More details are presented at https://www.youtube.com/watch?v=QKZu46c4HfU&feature=youtu.be.

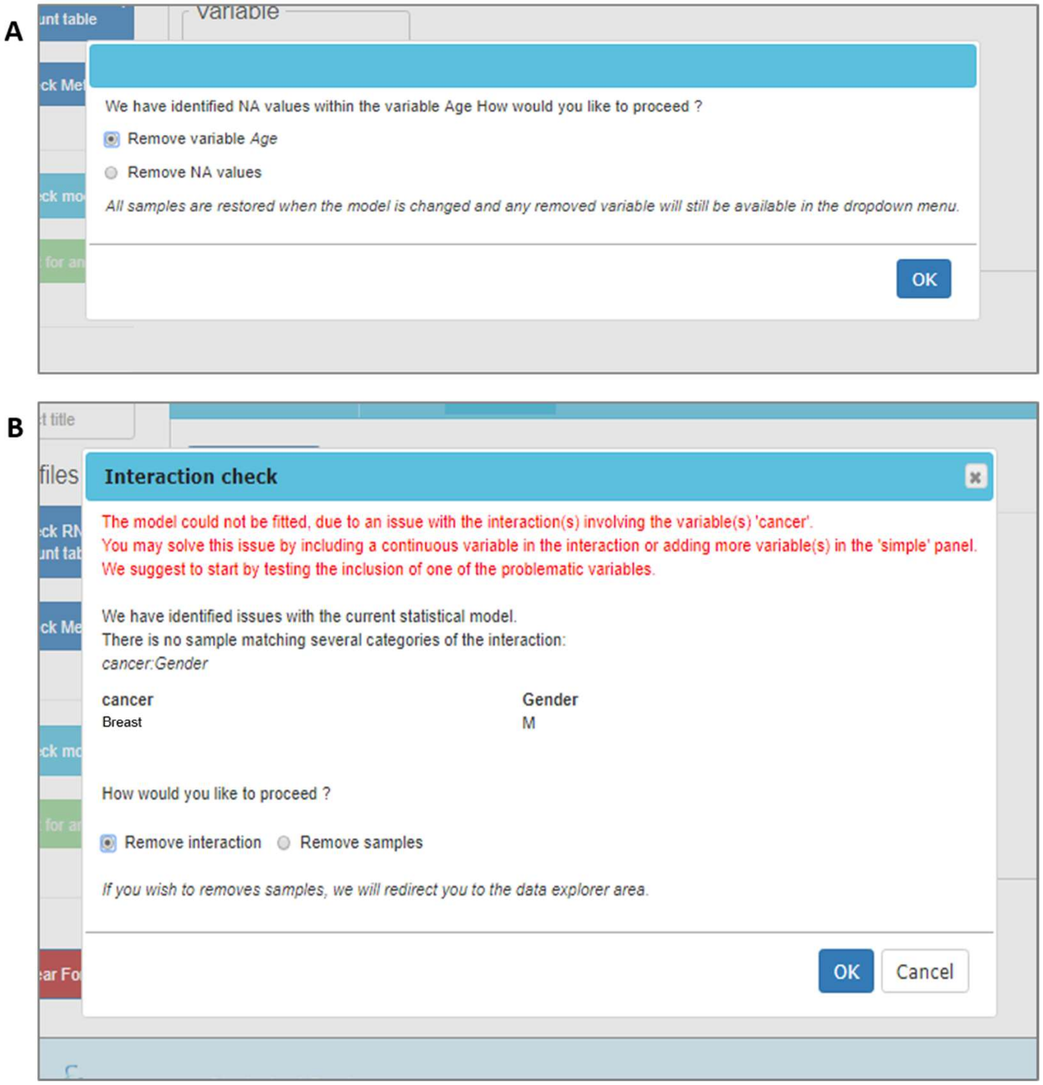

Supplementary Figure 2. Data validation. **(A)** The software can detect inconsistencies on the data upload and **(B)** when checking the model fitness.

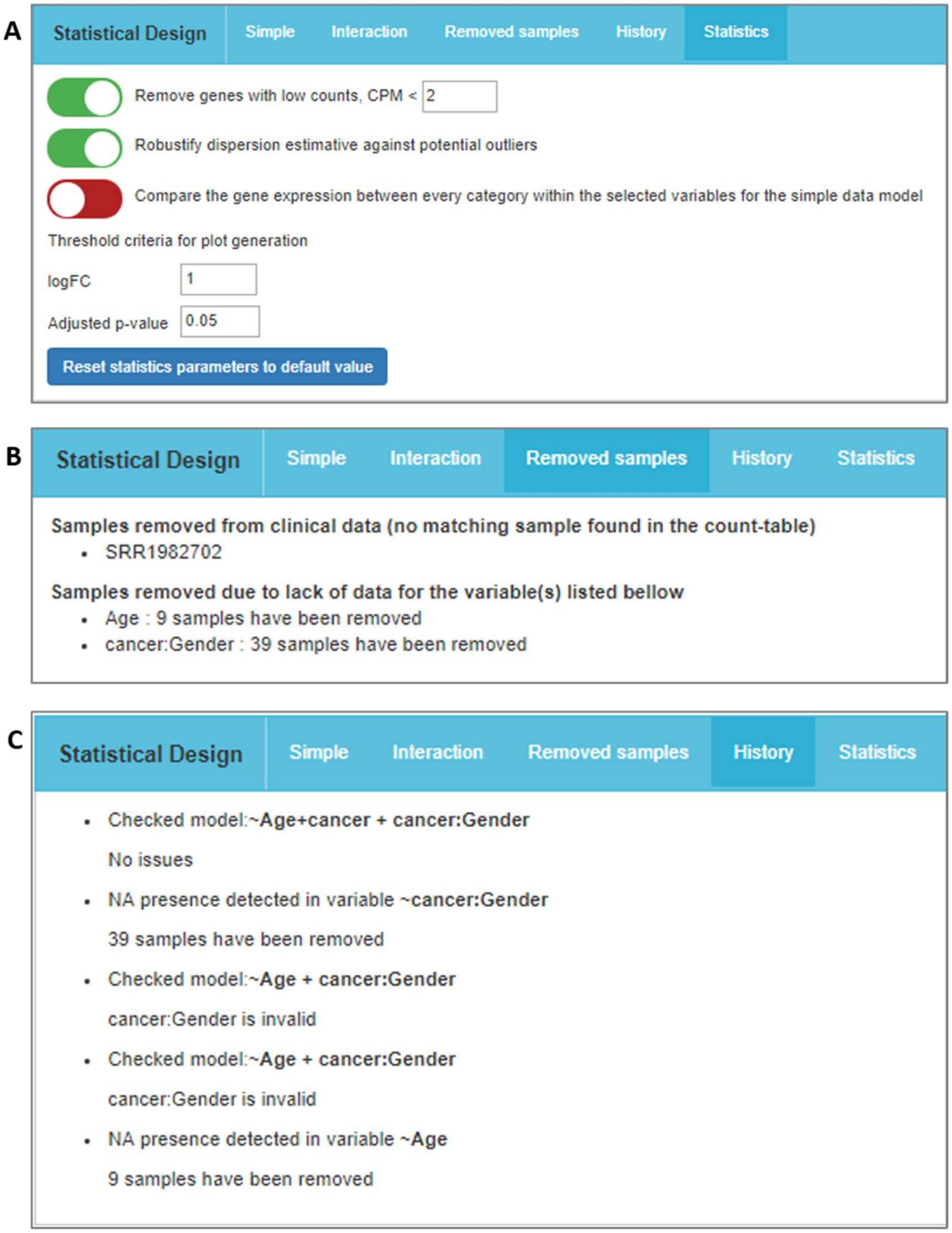

Supplementary Figure 3. Other features. **(A)** It is possible to choose the settings for filtering genes, statistical methods and the criteria for considering a gene differentially expressed. **(B)** The interface keeps track of the samples that needed to be removed and **(C)** tracks the tested models and changes done.

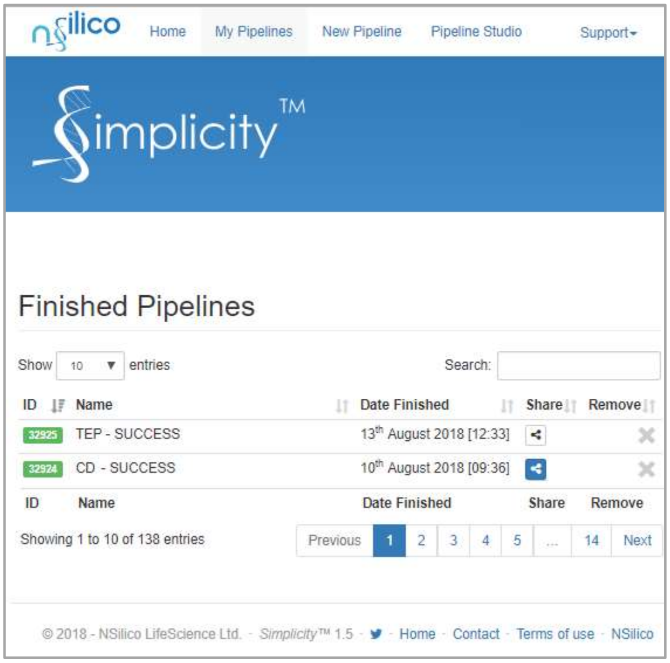

Supplementary Figure 4. *Simplicity*'s *'My Pipelines'* menu. In this page, all submitted pipelines are listed. The pipeline ID is followed by the Project Title and completion status, with the finish date and the option to share the results with other users and delete the pipeline.
